# Thyroid hormone receptor α1 acts as a new squamous cell lung cancer diagnostic marker and poor prognosis predictor

**DOI:** 10.1038/s41598-021-86754-6

**Published:** 2021-04-12

**Authors:** Fatma El Zahraa A. Mohamed, Ali Omar Abdelaziz, Ahmed Hussein Kasem, Tarek Ellethy, Mariana F. Gayyed

**Affiliations:** 1 Pathology department, Faculty of Medicine, Minia University, Minia, Egypt; 2 Chest department, Minia University Hospital, Minia University, Minia, Egypt; 3 Department of Radiotherapy and Nuclear Medicine, South Egypt Cancer Institute, Assiut University, Assiut, Egypt; 4 Department of Radiation Oncology, Katharinen Hospital, Stuttgart, Germany

**Keywords:** Oncology, Pathogenesis

## Abstract

Lung cancer is considered the major cause of cancer-related deaths worldwide. Unfortunately, all chemotherapy regimens used in lung cancer treatment showed nearly the same efficacy. Finding a new therapeutic target that can be used as an alternative after the failure of or in association with chemotherapy to improve the prognosis is an urgent demand. Up to date, it is Known that thyroid hormones (THs) and Thyroid hormone receptors (THRs) control the progression of several types of tumours. Nevertheless, their role in non-small cell lung cancer (NSCLC) is unknown. This study investigated the expression of THRα1 in NSCLC cases and its correlation to tumour clinicopathological parameters to shed new light on the relevance of THRα1 in lung cancer. Immunohistochemistry utilizing THRα1 antibody was performed on tissue sections obtained from 80 patients diagnosed with NSCLC. We also investigated the expression of THRα gene in Microarrays of lung squamous cell carcinoma (SCC) and adenocarcinoma (AC) patients by using GEO data sets on https://www.ncbi.nlm.nih.gov. We showed, for the first time, the expression of THRα1 in NSCLC. Intermediate and high THRα1 expressions were detected in (25% and 66.7%) of SCC cases respectively. High THRα1 expression was associated with shorter OS. On the other hand, 86.7% of AC cases revealed low THRα1 expression. Inflammatory cells in SCC cases showed high THRα1 expression. By analysing GEO data sets, a significant increase in THRα gene expression was found in SCC compared to AC cases. Our study underscores the possibility of using THRα1 expression not only as a prognostic marker, but also as an innovative diagnostic additive tool for lung SCC, which could be tested as a potential therapeutic target for SCC in the future.

## Introduction

Lung cancer is considered the first cause of cancer-related deaths worldwide. It is known as a highly invasive tumor and rapidly metastasizing cancer. Non-small cell lung carcinoma (NSCLC) forms about 85% of lung cancer cases^1^ of which squamous cell carcinoma (SCC) and adenocarcinoma (AC) are the most common types. Lung carcinogenesis is recognized to be a heterogeneous process, which occurs due to serious molecular genetic alterations^2^. Although the management lines of both tumors are similar, there is diversity in the genetic background of SCC and AC. Therefore, identifying tumor specific signatures by defining the altered genes can help in the early detection of NSCLC^3^ and innovate a tailored targeted therapy. For instance, in lung AC there is an amplification of proto-oncogenes such as MET, whereas in SCC others like fibroblast growth factor receptor1 (FGFR1) and Discoidin domain-containing receptor 2 (DDR2) are amplified. Thus, pemetrexed maintenance therapy is effective in AC patients but not in SCC patients, whereas docetaxel treatment is effective in SCC patients^4^.

Sadly, over the last three decades, the incidence of lung cancer has increased and the 5-year survival for diagnosed patients during 2010–2014 was unsatisfying^5^. Recently, numerous agents were introduced aiming at better disease control and prolonged patient survival. Unfortunately, all chemotherapy regimens used in lung cancer treatment showed nearly the same efficacy and the median overall survival (OS) is 7.9 months^6^. Finding a new therapeutic target that can be used as an alternative after the failure of or in association with chemotherapy to improve the tumor response and prognosis is currently an urgent demand and consequently a very interesting area of research. It is already known that the thyroid hormone (TH) has a pivotal role in several vital processes in human tissue. Additionally, its abnormal signaling was found to underlie many diseases^7–9^. The pathogenesis and relation between thyroid hormones and cancer is now better understood especially after the discovery of a non-genomic pathway for TH action. αvβ3 is a plasma membrane integrin which acts as a membrane receptor for TH^10^. This receptor has two specific binding sites for the hormones, S1 and S2, both of them can translate unique signaling cascades^11^. The S1 site binds exclusively physiological levels of T3, which in turn activates PI3K. The S2 site can bind binds T4 as well as with a lower affinity T3 leading to activation of the ERK1/2 pathway^12^. In this way, αvβ3 integrin binding promotes the hormones’ proliferative effect on cancer cells through a non-genomic pathway. Regarding the lung cancer model, increased expression of markers of cell proliferation like proliferating cell nuclear antigen (PCNA) and ERK1/2 activation which play an important role in the pathogenesis of lung cancer is associated with increased expression of T3 and/or T4. So hyperthyroidism enhance tumor growth and angiogenesis, while hypothyroidism suppress tumor proliferation^13^. While thyroid hormones promote phosphorylation of estrogen receptor α (ERα), an ERα antagonist blocked T4 induced PCNA expression, ERK1/2 activation and hence ERα phosphorylation. This means that the mitogenic effect of thyroid hormone mediated via the plasma membrane may involve an ERα dependent pathway. Tetrac, as well as pharmacologic inhibition of the MAPK pathway, suppressed proliferation of lung cancer cells in response to thyroid hormones^13,14^. Furthermore, in NSCLC, physiological concentrations of T4 facilitated internalization as well as nuclear translocation of the integrin αv monomer that binds inside the cell nucleus promoters of central cancer-related genes, such as thyroid hormone receptor β1, ERα and cyclooxygenase-2 and hypoxia-inducible factor-1α (HIF1α)^15^. Furthermore, the thyroid receptor-interacting protein 13 TRIP13 gene is amplified in early-stage non-small cell lung cancer^3^.

Therefore, TRIP13 was defined as a tumor promoter in NSCLC, which is responsible for regulating cell proliferation and invasion and its overexpression in lung cancer is associated with poor prognosis^16^. It has already been established that the thyroid hormones T3 and T4 modulate cancer hallmarks including cell proliferation, cell apoptosis, new angiogenesis, and invasiveness of cancer^17^. Thyroid hormones perform their action through thyroid hormone receptors (THRs) that act as ligand-dependent transcription factors^18^. T3 mediates metabolic activity by forming a complex between T3 and nuclear thyroid hormone receptors alpha (THRα). This nuclear T3-receptor complex binds to thyroid hormone response elements on specific genes and regulates their transcription (genomic pathway)^10^. In short, there are two isoforms of THRα (1 and 2). THRα1 can bind to T3 and subsequently promotes its effects, whereas THRα2 lacks this binding site leading to a different function than THRα1^19^. THRα2 regulates the isoform α1^20^. Moreover, previous studies show that THRα2 can act as a THRα1 antagonist^21^. Despite the paucity of information regarding THRα1 expression and its role in tumours, Jerzak and colleagues reported that high THRα1 expression was detected in about 70% of breast cancer samples. This high expression was associated with recurrence free survival. In addition, a significant association was revealed between the expression of THRα1, tumour size and tumour stage^19^.

It was observed that the Thyroid Hormone receptor α mRNA increases during the fetal lung development from early stage to late stage^22^ suggesting that this receptor is involved in the process of development of the lung and may have an influence on tumor development as well. Up to date, it is believed that THRα controls many tumors progression such as breast^19^, nevertheless, its role in NSCLC is not yet clear. All the above mentioned data provide evidence that TH and THRα may influence tumour behaviour. Therefore, in this study, we aim to investigate the expression of THRα1 in NSCLC cases. Additionally, we explore whether its expression correlates with patient clinicopathological parameters. Our results could improve the diagnostic and consequently the prognostic evaluation of patients with NSCLC.

## Patients and methods

### Patients

A retrospective study was performed on 80 patients diagnosed with non-small cell lung carcinoma; 48 cases were squamous cell carcinoma and 32 were adenocarcinoma. The cases were diagnosed and treated at Minia Oncology Center and Minia University Hospital, Egypt, during the period between January 2015 and December 2018. Only cases with available adequate tumor tissue and complete clinicopathological data were considered eligible. These Patients did not receive neoadjuvant therapy. The diagnosis was done by bronchoscopic evaluation of patients presenting with dyspnea, cough, hemoptysis, and chest pain and confirmed histopathological after bronchoscopic sample. For comparison, 15 sections of adjacent non-tumor lung tissue from different patients were also examined, in addition to 4 cases of normal lung tissue. Clinical data were obtained from the pathology reports and medical records and included the age of patients which ranged from 40 to 77 years with a mean (± standard deviation: SD) of 56.65 (± 1.12) years and a median of 55 years. NSCLC types and other patients’ clinicopathological data were shown in Table 1. A written informed consent was obtained from all patients at the time of hospital admission for biopsy. All methods were carried out in accordance with relevant guidelines and regulations. All experimental protocols were approved by Minia Oncology Center and Minia University Hospital ethical committee.

**Table 1:** The clinicopathological characteristics of NSCLC cases (No = 80).

Clinicopathological characteristics	SCC (48 cases)	Adenocarcinoma (32 cases)
	No (%)	No (%)
**Age (y)**		
≤ 55	12 (25)	32 (100)
> 55	36 (75)	0
**Gender**		
Male	38 (79.2)	20 (62.5)
Female	10 (20.8)	12 (37.5)
**Tumor Grade**		
GI	6 (12.5)	7 (21.9)
GII	23 (47.9)	12 (37.5)
GIII	19 (39.6)	13 (40.6)
**T Stage**		
T1	4 (8.3)	12 (37.5)
T2	30 (62.5)	8 (25)
T3	14 (29.2)	12 (37.5)
**N Stage**		
N0	21 (43.8)	17 (53.1)
N1	13 (27.1)	13 (40.6)
N2	14 (29.2)	2 (6.2)
**Metastasis**		
M0	37 (77.1)	31 (96.9)
M1	11 (22.9)	1 (3.1)
**TNM Stage**		
I	15 (31.2)	12 (37.5)
II	15 (31.2)	3 (10)
III	7 (14.6)	3 (10)
IV	11 (22.9)	0
**Survival**		
Event	35 (72.9)	3 (9.4)
Censored	13 (27.1)	29 (90.6)
**THRα expression**		
Low	4 (8.3)	28 (87.5)
Intermediate	12 (25)	3 (9.4)
High	32 (66.7)	1 (3.1)

### Immunohistochemistry

Immunohistochemistry (IHC) was performed on 4-μm tissue sections taken from 10% buffered formalin-fixed, paraffin-embedded tissue blocks. The IHC was performed using an automated immune-stainer (Ventana Bench-Mark GX; Ventana Inc.) according to manufacturer recommendation. Antigen retrieval was carried out utilizing Tris-based reaction buffer concentrate (pH 7.6). Rabbit anti-human Thyroid Receptor Alpha polyclonal antibody isoform 1 (1/50 dilution, MyBioSource, USA), mouse monoclonal P63 (prediluted, Roche, Germany), Napsin A (prediluted, Roche, Germany) were used as primary antibodies for 32 min, then the visualization was performed by Avidin–Biotin detection system. A case of breast cancer was used as a positive control for THRα1. Negative controls were achieved by omitting the primary antibody.

### Evaluation of immunohistochemical staining

The specimens were evaluated independently by two of the authors (M.G. & F.M.) in a blind fashion to the clinicopathological data. The cytoplasmic THRα immunoreactivity in cells was evaluated by considering the intensity and percentage of staining as follows: Allred’s method^16,23^, was tailored to score each of the immunohistochemically stained sections for THRα1; scores for the intensity of staining (absent: 0, weak: 1, moderate: 2 and strong: 3) were added to the percentage of cells stained (none: 0–1%, 1: 1–10%, 2: 11–33%, 3: 34–66%, and 4:67–100%) to range a score of 1–8. Finally, a score of 1–3 was considered low and a score of 4–6 intermediate and 7–8 was deemed high. Nuclear expression of p63 and cytoplasmic expression of Napsin A were considered positive.

### Data set analysis

We investigated the expression of thyroid hormone receptor α gene (named in the data set as THRA) values in SCC and AD patients from previous gene microarray studies by using Gene Expression Omnibus database (GEO) data sets on https://www.ncbi.nlm.nih.gov. The values of this gene were extracted from every data set by selecting two groups -e.g. SCC and AD, then the value of the gene in the microarray was calculated after pressing save results as described in a previous publication^24^. The following data sets were investigated:

- $$\mathrm{GSE}1115457\,\,\,\text{ SCC n}= 15\,\,\,\mathrm{and}\,\,\, AC \,\,n= 20$$
- $$G\mathrm{SE}19188\,\,\,\text{ SCC n}= 27\,\,\,\mathrm{and}\,\,\, AC\,\, n= 45$$
- $$\mathrm{GSE}7880\,\,\,\text{ SCC n}= 10\,\,\,\mathrm{and}\,\,\, AC \,\,\,n= 10$$

### Statistical analysis

All statistical analyses were performed using SPSS 20.0 computer software. To test associations between categorical variables, Chi-square and Fisher exact tests were conducted. The effect of THRα on the prognosis of NSCLC patients was assessed via univariate and multivariate Cox regression. Hazard risk (HR) and relative 95% confidence interval (CI) were analyzed. P of 0.05 was used as a significance criterion.

## Results

### THRα1 immunoreactivity in NSCLC

**Figure 1:**
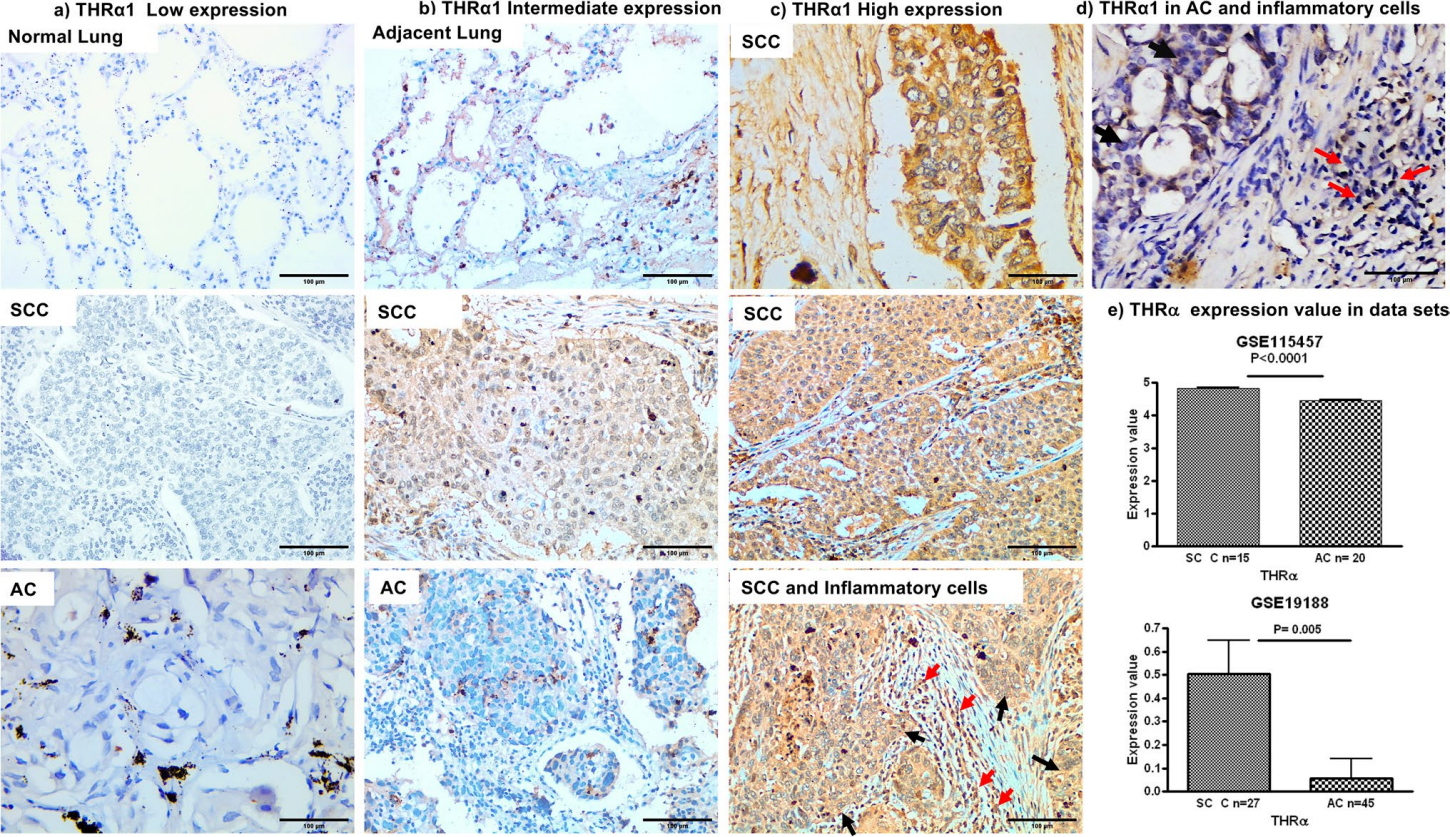
THRα1 protein expression in human NSCLC tissues and gene expression in GEO data sets. (**a**) low THRα1 protein expression in normal lung tissue, SCC, and AC. (**b**) Intermediate THRα1 protein expression in adjacent lung tissue, in SCC and in AC. (**c**) High THRα1 protein expression in most SCC tumour cells and associated inflammatory cells. (**d**) Low THRα1 protein expression in AC tumour cells, no THRα1 protein expression in associated inflammatory cells. Magnification 200x, scale bar 100 µm, black arrows pointed to tumour cells and red arrows to inflammatory cells. (**e**) THRα mRNA expression levels in data sets showing significant high expression in SCC compared to AC.

THRα1 expression was detected in the cytoplasm of NSCLC but not in normal lung tissue as shown in (Fig. 1). THRα1 was detected in lung tissue adjacent to tumor, the expression level ranges from low to intermediate expression. In our cases, it was noticed that THRα1 expression was clearly shown in the majority of SCC compared to AC cases. About 91.6% of SCC showed either intermediate or high expression, whereas 87.5% of AC cases showed low THRα1 expression (*p* = 0.001). Interestingly, inflammatory cells in SCC cases showed high THRα1 expression even in cases with intermediate expression. No inflammatory cells in AC cases showed high THRα1 expression.

### THRα1 acts as a diagnostic marker for SCC in poorly differentiated lung carcinoma cases

**Figure 2:**
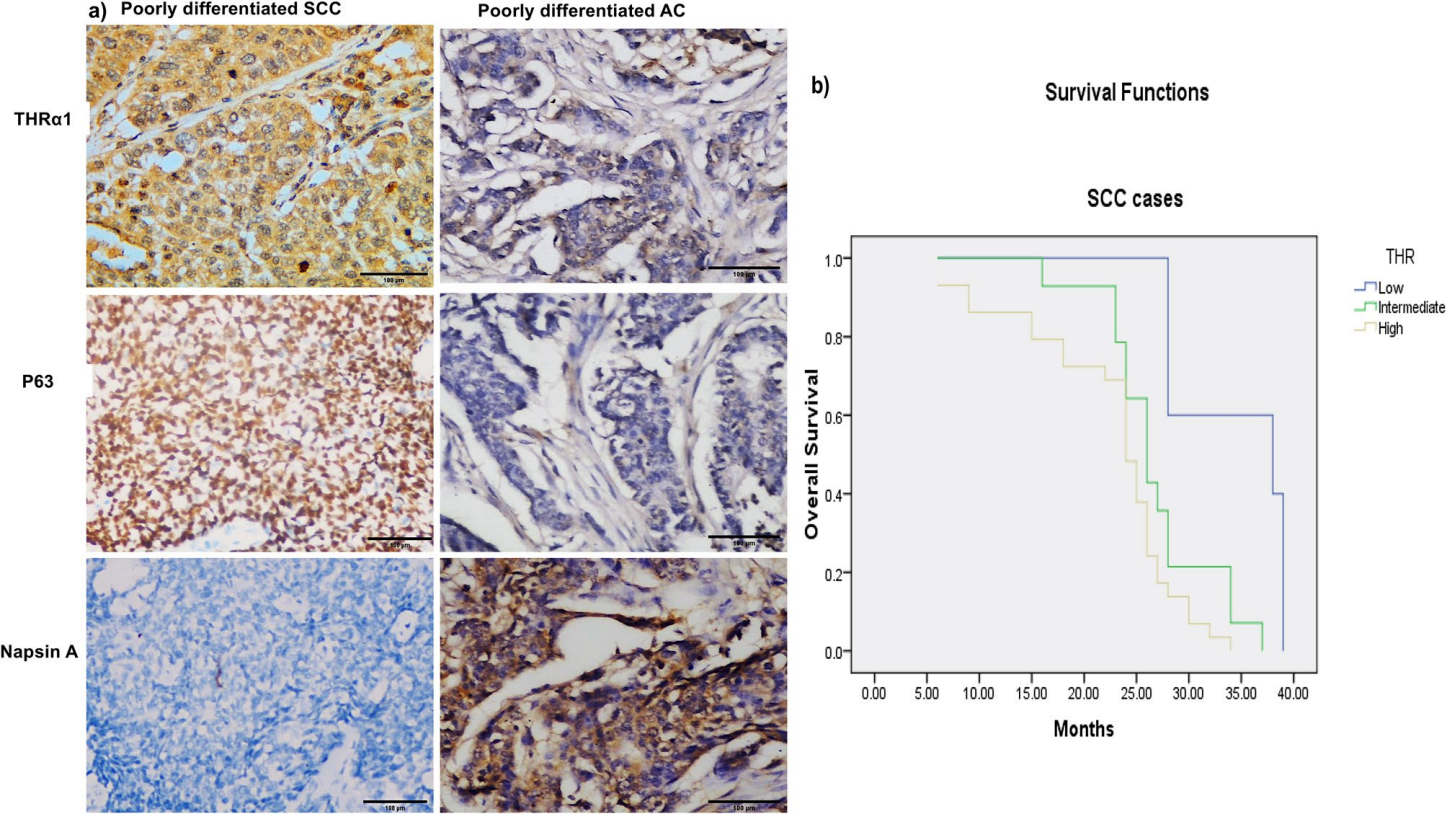
High THRα1 expression is a diagnostic marker for SCC in poorly differentiated human NSCLC tissues and is associated with shorter OS. (**a**) Poorly differentiated NSCLC tissues showing high THRα1 protein expression, positive nuclear P63 and negative Napsin A confirming that the case is SCC and low THRα1 protein expression, negative nuclear P63 and positive Napsin A confirming that the case is AC. **(b**) Kaplan Meyer curve showing that THRα1 expression is associated with poor OS.

In our study, by investigating the 32 cases which were diagnosed as poorly differentiated NSCLC, 16 (50%) cases showed high THRα1 expression whereas, 10 (31%) cases revealed low expression. Interestingly, 15 (93.7%) out of these 16 cases revealed positive P63 expression—the diagnostic SCC marker- and negative Napsin A—the AC marker- (Fig. 2). This finding confirmed that the poorly differentiated NSCLC cases with high THRα1 were originally SCC and pointed to THRα1 as a potentially additive diagnostic marker.

On the other hand, All the 10 cases showed low expression were adenocarcinoma confirmed by Napsin A staining (Fig. 2).

### In silico study

By analyzing the in-silico data, the expression of THRα gene was significantly higher in SCC compared to AC in the following data sets GSE115457, GSE19188 *p* = 0.0001 and 0.005 respectively (Fig. 1). Although the variation did not reach the significant level in GSE7880, the trend of increase in the expression in SCC than AC still exists.

### THRα1 is a prognostic marker in SCC cases

#### THRα1 expression in SCC cases

The expression was detected high in 32 (66%) cases, intermediate in 12 (25%), and low in 4 (8.3%). A significant association was noticed between increasing tumor grade and high expression (*p* = 0.011). Regarding the tumor stage, a significant relationship was found between increased THRα1 expression and T stage N stage, Metastasis, and TNM stage (*p* = 0.001, *p* = 0.007, *p* = 0.017, and *p* = 0.003 respectively). No significant association was revealed with age or gender. Table 2.

**Table 2:** The association between THRα1 expression and clinicopathological characteristics of NSCLC (SCC) cases (No = 48).

Clinicopathological characteristics	THRα expression			*p* value
	Low (%)	Intermediate (%)	High (%)	
**Age**				
≤ 55	2 (16.7)	4 (33.3)	6 (50)	0.232
> 55	2 (5.6)	8 (22.2)	26 (72.2)	
**Gender**				
Male	4 (10.5)	9 (23.7)	25 (65.8)	0.742
Female	0 (0)	3 (30)	7 (70)	
**Tumor grade**				
GI	3 (50)	0 (0)	3 (50)	0.011*
GII	1 (4.3)	8 (34.8)	14 (60.9)	
GIII	0 (0)	4 (21.1)	15 (78.9)	
**T stage**				
T1	2 (50)	0 (0)	2 (50)	0.001*
T2	2 (6.7)	12 (40)	16 (53.3)	
T3	0 (0)	0 (0)	14 (100)	
**N stage**				
N0	2 (9.5)	9 (42.9)	10 (47.6)	0.007*
N1	2 (15.4)	3 (23.1)	8 (61.5)	
N2	0 (0)	0 (0)	14 (100)	
**Metastasis**				
M0	4 (10.8)	12 (32.4)	21 (56.8)	0.017*
M1	0 (0)	0 (0)	11 (100)	
**TNM stage**				
Stage I	2 (13.3)	8 (53.3)	5 (33.3)	0.003*
Stage II	2 (13.3)	4 (26.7)	9 (60)	
Stage III	0 (0)	0 (0)	7 (100)	
Stage IV	0 (0)	0 (0)	11 (100)	
**Overall survival**				
Censored	3 (23.1)	6 (46.2)	4 (30.8)	0.003*
Event	1 (2.9)	6 (17.1)	28 (80)	

Test of significance: Chi-square and Fisher exact tests.

*p*-value < 0.05 is considered significant.

#### THRα1 expression in AC cases

The expression of THRα1 was detected high in only 1 (3.1%.) case, intermediate in 3 (9.4%), and low in 28 (87.5%) cases. Therefore, it was not surprising to find no significant association between the expression and any clinicopathological parameters Table 3.

**Table 3:** The association between THRα1 expression and clinicopathological characteristics of NSCLC (AC) cases (No = 32).

Clinicopathological characteristics	THRα expression			*p* value
	Low (%)	Intermediate (%)	High (%)	
**Age**				
≤ 55	26 (86.7)	3 (10)	1 (3.3)	0.573
> 55	2 (100)	(0)	(0)	
**Gender**				
Male	18 (90)	1 (5)	1 (5)	0.715
Female	10 (83.3)	2 (16.7)	0 (0)	
**Tumor grade**				
GI	6 (85.7)	1 (14.3)	0 (0)	0.414
GII	12 (100)	0 (0)	0 (0)	
GIII	10 (76.9)	2 (15.4)	1 (7.7)	
**T Stage**				
T1	12 (100)	0 (0)	0 (0)	0.231
T2	6 (75)	1 (12.5)	1 (12.5)	
T3	10 (83.3)	2 (16.7)	0 (0)	
**N Stage**				
N0	16 (94.1)	0 (0)	1 (5.9)	0.005*
N1	12 (92.3)	1 (7.7)	0 (0)	
N2	0 (0)	2 (100)	0 (0)	
**Metastasis**				
M0	28 (90.3)	2 (6.5)	1 (3.2)	0.125
M1	0 (0)	1 (100)	0 (0)	
**TNM stage**				
Stage I	12 (100)	0 (0)	0 (0)	0.012*
Stage II	10 (100)	0 (0)	0 (0)	
Stage III	6 (60)	3 (30)	1 (10)	
Stage IV	0 (0)	0 (0)	0 (0)	
**Overall survival**				
Censored	28 (96.6)	1 (3.4)	0 (0)	0.001*
Event	0 (0)	2 (66.7)	1 (33.3)	

Test of significance: Chi-square and Fisher exact tests.

*p*-value < 0.05 is considered significant.

### Survival analysis

The follow-up was ranged from 6 to 39 months with a mean of 25.85 (± 6.7) months and a median of 26 months for SCC cases. For AC cases it was ranged from 24 to 42 months with a mean of 34.9 (± 3.9) months and a median of 35 months. Regarding marker expression and OS, increased expression of THRα1 was associated with worse OS (*p* = 0.002) for SCC cases (Fig. 2). On the other hand, the opposite was found in AC cases; low expression was associated with prolonged survival.

The relationship between the prognosis and the expression of THRα1 in SCC cases was evaluated via univariate and multivariate Cox regression. The univariate regression, Table 4 indicated grade (grade III versus II: HR = 2.14, 95%, CI: 2.01–35.9, *p* = 0.004), TNM stage (stage III: HR = 1.83, 95%, CI: 1.34–29.2, *p* = 0.019), and high THRα1 expression (HR = 1.5, 95%, CI: 1.18–17.7, *p* = 0.027) were all associated with poor survival status of SCC patients. The multivariate regression, Table 5 stated positive THRα1 expression significantly increased the risk of adverse consequences and poor prognosis (HR = 0.86, 95%, CI: 1.1–5.06, *p* = 0.025).

**Table 4:** Univariate Cox regression analysis of relationship between clinicopathological characteristics and prognosis in cases with NSCLC (SCC).

Type	B	SE	Wald	Sig	Exp(B)	95.0% CI for Exp (B)	
						Lower	Upper
**NSCLC (SCC)**							
Age	− 1.282	0.780	2.704	0.100	0.277	0.060	1.279
Sex	− 5.200	1.166	19.884	0.000	0.006	0.001	0.054
Grade			9.437	0.009			
Grade (1)	− .523	1.454	0.129	0.719	0.593	0.034	10.249
Grade (2)	2.140	0.735	8.469	0.004	8.499	2.011	35.916
Stage			9.199	0.027			
Stage (1)	− 1.837	1.565	1.378	0.240	0.159	0.007	3.421
Stage (2)	− 2.728	1.319	4.277	0.039	0.065	0.005	0.867
Stage (3)	1.837	0.785	5.467	0.019	6.275	1.346	29.254
T			22.336	0.000			
T (1)	− 3.293	1.620	4.129	0.042	0.037	0.002	0.890
T (2)	− 4.656	1.061	19.262	0.000	0.010	0.001	0.076
N			9.523	0.009			
N (1)	− 2.926	1.486	3.879	0.049	0.054	0.003	0.986
N (2)	0.733	1.101	0.444	0.505	2.082	0.241	18.000
M			–	–			
THR			4.881	0.087			
THR (1)	0.518	2.056	0.064	0.801	1.679	0.030	94.362
THR (2)	1.525	0.690	4.877	0.027	4.594	1.187	17.777

**Table 5:** Multivariate Cox regression analysis of relationship between clinicopathological characteristics and prognosis in NSCLC (SCC) cases.

Type	B	SE	Wald	Sig	Exp(B)	95.0% CI for Exp (B)	
						Lower	Upper
**NSCLC (SCC)**							
Grade	− 0.0422	0.310	1.847	0.174	0.656	0.357	1.205
Stage	0.526	0.178	8.774	0.003	1.692	1.195	2.396
THR	0.867	0.385	5.056	0.025	2.379	1.118	5.062

## Discussion

Lung cancer is a highly aggressive disease with high morbidity and mortality and the 5-year survival is unsatisfactory despite the application of updated therapeutic protocols. It was reported that Thyroid-stimulating hormone receptor (TSHR) binds to NK2 homeobox1 (NKX2-1), the previously characterized lung cancer marker^25^. TSH binds to TSHR to stimulate the production of T3, the latter binds to THRα1 to mediate metabolic activities via the genomic pathway. This cascade suggests an indirect connection between TSHR and THRα1.

Also, in lung cancer cells, proliferating cell nuclear antigen (PCNA) expression and extracellular signal-regulated kinases 1/2 (ERK1/2) activation were induced upon THs treatment^13^, suggesting the possible role of THs and their receptors in lung cancer progression. This was noticed particularly after realizing that thyroid hormones and THRs are implicated in the pathogenesis of many tumors such as breast^19^, prostate^26^, ovary^27^, colon^28^ and Hepatoma^29^. However, the role of THR in NSCLC is not fully understood until now.

In this study, we investigated the expression of THRα1 in NSCLC in order to evaluate whether its expression adds to the prognostic parameters, and hence a better prognostic evaluation. Furthermore, the variable expression of THRα1 in many tumors^26–29^ raises our interest and sheds new light on its possible wide therapeutic value. The possibility of using THRα1 as a target therapy was discussed in a previous breast cancer study^19^.

A careful analysis of our sections revealed that THRα1 expression was found in the cytoplasm of NSCLC. Interestingly, high and intermediate expression was shown mainly in 91% of SCC cases compared to AC cases which showed low expression in 87% of cases. In line with our finding, cytoplasmic expression of THRα1 was shown in 74% breast cancer cases and authors suggested that thyroid hormones promote tumor growth^19^. Supporting our results, investigating data sets showed a significant increase in THRα mRNA expression level in SCC cases in GSE115457, GSE19188. Notably, poorly differentiated cases which showed high THRα1 expression, were positive for P63 and negative for Napsin A, suggesting that these cases should be diagnosed as lung-SCC. This observation raises the possibility for using THRα1 as one of the diagnostic markers for lung-SCC.

The tumor microenvironment is vital in NSCLC management as lymphocytic infiltration and macrophages were reported to promote or suppress tumor progression^30^. Moreover, immune therapy is recently being used in NSCLC treatment, and finding predictive parameters was a target for several years. Up to date, focusing on PD-L1 expression is used for a specific therapy target^31^. In this study, inflammatory cells infiltrating lung-SCC showed high THRα1 expression. Therefore, THRα1 expression in inflammatory cells may serve a potential target in immune therapy.

Further evaluation of THRα1 expression in lung-SCC cases revealed a significant positive association between increased THRα1 expression and tumor grade. The shortage of THRα studies persuaded the tracing of TH effect on other tissues to understand THR behavior in cancer. A previous study showed that TH correlates with SCC grades and is associated with shorter disease-free survival^32^. In Jerzak’s study, the median expression of THRα1 and THRα2 in samples from a cohort of breast cancer patients was assessed based on the Allred score, with a range from 0 to 8. The median expression of THRα1 was 7 and was not associated with tumor size, grade or stage of disease as a continuous variable. Although the median expression of THRα2 was 5, also as a continuous variable was not associated with Tumor stage or grade but high expression of THRα2 was associated with an improved overall survival [HR 0.29 95% CI (0.10–0.85), *p* = 0.024]. The study concluded that lowered THRα1 expression is associated with improved 5-year survival. Moreover, THRα1 expression was proven to influence tumor growth in their samples, which is consistent with our finding^19^. THRα1 may be responsible for the development of tumor but not its differentiation as in the case of the zebrafish model where THRα1, not THRα2, is responsible for early embryonic development^33^. THRα is known to be associated with antiapoptotic effects as well as maintaining survival and protecting pancreatic cells from ER stress^34^. Perhaps a similar mechanism occurs during cancer progression, protects cancer cells from apoptosis, and maintains its progression.

In our lung-SCC cases, high THRα1 was positively associated with T stage, N stage, metastasis and TNM stage. In line with our findings, a significant association was detected between high THRα1 and increasing breast cancer TNM stage and T stage^19^. Another study reported that cases with lympho-vascular invasion showed higher THRα1 expression compared to cases without invasion^35^. These results support the concept of involving THRα1 in tumor progression particularly metastasis and invasion. Backing this thought experimentally, in the Lewis lung cancer model, which is a model of poorly differentiated lung squamous cell carcinoma, treatment with THs induces tumor progression and metastasis. On the other hand, induced hypothyroidism using methimazole is associated with attenuation tumor growth and significantly reduces tumor metastasis and prolongs survival^17,36^. This indicates that SCC relies on TH and subsequently THR for tumor progression and metastasis. It was reported that increased thyroid hormone (TH) levels promote the epithelial-mesenchymal transition and malignant progression of SCC cells by upregulating ZEB-1, mesenchymal genes and metalloproteases as well as suppressing E-cadherin expression^32^. The fore-mentioned theories in the previous studies can explain that THRα1 is upregulated in SCC to control its invasion.

In lung-SCC cases, higher THRα1 expression was significantly associated with shorter OS and it was confirmed to be an independent poor prognostic marker according to the multivariate analysis, whereas the opposite occurs in AC cases. However, the fact that most AC cases had low THRα1 expression makes it hard to decipher whether low expression in AC is significantly associated with better OS.

Due to the insufficiency of information regarding THRα1 in NSCLC another research was done on the available data on scientific websites. According to the protein atlas website, THRα was expressed in lung cancer cases with moderate intensity. Moreover, the expression was detected in the cytoplasm as well as in the cell membranes of both SCC and AD. The controversy between these results and ours may be attributed to the used antibody (CAB023349), which is specific for THRα2 isoform, whereas in our study THRα1 was used^37^. In conclusion, although our study has some limitations including patient numbers and deep investigation of THRα1 expression in inflammatory cells. We hereby showed, for the first time, the expression of THRα1 in NSCLC. In our cases, high THRα1 expression was detected in most of squamous cell lung cancer cases and was associated with shorter OS and poor prognostic parameters. These findings not only point to the potential use of THRα1 as a prognostic marker, but also shed new light on its diagnostic value in lung-SCC. Further investigation on its value as a therapeutic target is recommended.
